# *Restorer of fertility like 30*, encoding a mitochondrion-localized pentatricopeptide repeat protein, regulates wood formation in poplar

**DOI:** 10.1093/hr/uhae188

**Published:** 2024-07-15

**Authors:** Xiaokang Fu, Ziwei Yang, Li Guo, Lianjia Luo, Yuanxun Tao, Ting Lan, Jian Hu, Zeyu Li, Keming Luo, Changzheng Xu

**Affiliations:** Chongqing Key Laboratory of Plant Resource Conservation and Germplasm Innovation, Integrative Science Center of Germplasm Creation in Western China (Chongqing) Science City, School of Life Sciences, Southwest University, Chongqing 400715, China; Key Laboratory of Eco-environments of Three Gorges Reservoir Region, Ministry of Education, School of Life Sciences, Southwest University, Chongqing 400715, China; Chongqing Key Laboratory of Plant Resource Conservation and Germplasm Innovation, Integrative Science Center of Germplasm Creation in Western China (Chongqing) Science City, School of Life Sciences, Southwest University, Chongqing 400715, China; Key Laboratory of Eco-environments of Three Gorges Reservoir Region, Ministry of Education, School of Life Sciences, Southwest University, Chongqing 400715, China; Chongqing Key Laboratory of Plant Resource Conservation and Germplasm Innovation, Integrative Science Center of Germplasm Creation in Western China (Chongqing) Science City, School of Life Sciences, Southwest University, Chongqing 400715, China; Chongqing Key Laboratory of Plant Resource Conservation and Germplasm Innovation, Integrative Science Center of Germplasm Creation in Western China (Chongqing) Science City, School of Life Sciences, Southwest University, Chongqing 400715, China; Chongqing Key Laboratory of Plant Resource Conservation and Germplasm Innovation, Integrative Science Center of Germplasm Creation in Western China (Chongqing) Science City, School of Life Sciences, Southwest University, Chongqing 400715, China; Chongqing Key Laboratory of Plant Resource Conservation and Germplasm Innovation, Integrative Science Center of Germplasm Creation in Western China (Chongqing) Science City, School of Life Sciences, Southwest University, Chongqing 400715, China; Chongqing Key Laboratory of Plant Resource Conservation and Germplasm Innovation, Integrative Science Center of Germplasm Creation in Western China (Chongqing) Science City, School of Life Sciences, Southwest University, Chongqing 400715, China; Chongqing Key Laboratory of Plant Resource Conservation and Germplasm Innovation, Integrative Science Center of Germplasm Creation in Western China (Chongqing) Science City, School of Life Sciences, Southwest University, Chongqing 400715, China; Chongqing Key Laboratory of Plant Resource Conservation and Germplasm Innovation, Integrative Science Center of Germplasm Creation in Western China (Chongqing) Science City, School of Life Sciences, Southwest University, Chongqing 400715, China; Key Laboratory of Eco-environments of Three Gorges Reservoir Region, Ministry of Education, School of Life Sciences, Southwest University, Chongqing 400715, China; Chongqing Key Laboratory of Plant Resource Conservation and Germplasm Innovation, Integrative Science Center of Germplasm Creation in Western China (Chongqing) Science City, School of Life Sciences, Southwest University, Chongqing 400715, China; Key Laboratory of Eco-environments of Three Gorges Reservoir Region, Ministry of Education, School of Life Sciences, Southwest University, Chongqing 400715, China

## Abstract

Nuclear–mitochondrial communication is crucial for plant growth, particularly in the context of cytoplasmic male sterility (CMS) repair mechanisms linked to mitochondrial genome mutations. The *restorer of fertility-like* (*RFL*) genes, known for their role in CMS restoration, remain largely unexplored in plant development. In this study, we focused on the evolutionary relationship of *RFL* family genes in poplar specifically within the dioecious Salicaceae plants. *PtoRFL30* was identified to be preferentially expressed in stem vasculature, suggesting a distinct correlation with vascular cambium development. Transgenic poplar plants overexpressing *PtoRFL30* exhibited a profound inhibition of vascular cambial activity and xylem development. Conversely, RNA interference-mediated knockdown of *PtoRFL30* led to increased wood formation. Importantly, we revealed that *PtoRFL30* plays a crucial role in maintaining mitochondrial functional homeostasis. Treatment with mitochondrial activity inhibitors delayed wood development in *PtoRFL30*-RNAi transgenic plants. Further investigations unveiled significant variations in auxin accumulation levels within vascular tissues of *PtoRFL30-*transgenic plants. Wood development anomalies resulting from *PtoRFL30* overexpression and knockdown were rectified by NAA and NPA treatments, respectively. Our findings underscore the essential role of the PtoRFL30-mediated mitochondrion-auxin signaling module in wood formation, shedding light on the intricate nucleus–organelle communication during secondary vascular development.

## Introduction

Mitochondria, thought to have evolved from free-living bacterial ancestors absorbed by an archaeal-like host, have a separate genome [1]. During endosymbiotic evolution, most mitochondrial genes were lost or transferred to the cell nucleus [2]. Conversely, numerous proteins encoded by nuclear genes are essential for mitochondrial biogenesis and function [3]. Specifically, proteins destined for mitochondria actively participate in driving mitochondrial gene expression [4], forming the foundation for nucleus-dependent organelle control. Mitochondrial functionality dynamically modulates nuclear gene transcription, providing context-dependent feedback from mitochondria to the nucleus [5]. Bidirectional communication, termed anterograde and retrograde signaling, orchestrates interactions between mitochondrial and nuclear genomes [6], playing a crucial function in plant development and stress responses [5].

Cytoplasmic male sterility (CMS) and its restoration serve as an insightful model for probing nuclear–mitochondrial interactions in plant cells. The CMS phenotype is often linked to atypical chimeric open reading frames (ORFs) expressed in mitochondria [7]. These ORFs, arising from mitogenomic recombination and co-transcribed with normal mitochondrial genes, encode cytotoxic proteins that disrupt microsporogenesis and pollen viability [8]. Nuclear genes known as restorers of fertility (*Rf*) promote CMS restoration by normalizing pollen production in plants with sterile cytoplasm [9]. Among various plant species, like petunia, rice, rapeseed, and radish, identified *Rf* genes encode members of a large RNA-binding protein family characterized by tandem pentatricopeptide repeat (PPR) domains [10–12]. Initially discovered in *Arabidopsis thaliana* genomic sequences, the PPR protein family comprises 2–27 P-type 35-amino-acid domains (PPR domains) [10, 12]. Recent studies have identified two PPR domain variants: L-type domains (~35–36 amino acids) and S-type domains (31 amino acids) [13, 14]. Functionally, proteins containing P-type PPR domains are involved in 3′ and 5′ terminal processing, RNA stabilization, cleavage, translation activation, and RNA intron splicing regulation. In contrast, PLS-type PPR proteins are involved in C-to-U RNA editing [4, 15]. Specifically targeted to mitochondria, Rf-PPR proteins play a crucial role in recognizing and eliminating CMS-inducing transcripts, thereby restoring normal pollen production [16–19]. Altogether, Rf-PPR proteins emerge as pivotal mediators in orchestrating nuclear–mitochondrial interactions during CMS restoration.

The genomes of land plants contain a considerable expansion of the PPR protein family, but the Rf-PPR protein selection pattern is different from that of most other *PPR* gene families (*non-Rf*) [9, 20]. Most PPR proteins (non-Rf) undergo selection against mutations to conserve functional protein sequences [21]. Local sequence duplication is a common method used by plants to create new *Rf-PPR* genes in response to sterility-inducing genes [16, 17, 22]. Besides, Fujii *et al*. demonstrated that not only do members of the Rf-PPR protein family face diverse selection pressures, but different amino acids within a single Rf-PPR protein also face varying degrees of selection pressure [20]. The amino acids directly involved in CMS transcript recognition are 5–15 times more likely to be susceptible to varied selection than other amino acids in this domain [20, 23, 24]. Diversified selection of PPR proteins helps to more quickly and effectively create new Rf genes to silence newly emerging CMS transcripts, which is a successful strategy for dealing with conflicts between nuclear and mitochondrial genomes in angiosperm evolution.

Recently, mitochondrial-targeted members of the Rf-PPR family have emerged as crucial nuclear response factors for modifying mitochondrial homeostasis, playing indispensable roles in processes beyond CMS restoration during growth and development [25]. Rice mutants carrying *natural blight leaf* 3 (*nbl3*) not only exhibit delayed growth and premature senescence, but also demonstrate increased resistance to bacterial and fungal infections, as well as enhanced tolerance to salt stress [26]. Nine mitochondrial-targeted PPR proteins in *Arabidopsis* have been found to be involved in the plant’s responses to different abiotic or biotic stressors [27–34]. In maize, endosperm and embryonic development are almost completely halted due to the functional absence of *Small kernel* (*SMK*) genes [35–37]. Additionally, plants have evolved variable reproductive strategies. *Rf-PPR* genes are also present in the genomes of dioecious plants; however, their functions remain unclear [20]. According to our earlier studies, MiR476a specifically targets a number of *PPR* genes located in the mitochondria that are part of the *RFL* gene family. By altering the auxin pathway, MiR476a/RFL-mediated dynamic regulation of mitochondrial homeostasis affects adventitious root development [38]. At present, there is still no direct evidence that *Rf-PPR* genes are involved in wood formation, but recent research indicates that the vascular cambium region accumulates a large amount of reactive oxygen species (ROS), and the type (O_2_^−^ and H_2_O_2_) and level of ROS are key to maintaining cambium activity [39]. Mitochondria are one of the main production sites for ROS in plant cells, and mitochondrial homeostasis directly determines the formation levels of different ROS [40–43].These findings suggested that mitochondrial homeostasis may be necessary for plant vascular development. Therefore, there is a need to prove whether the Rf-PPR gene mediates mitochondrial homeostasis during wood formation in trees.

In this study, we identified 32 *RFL* genes in *Populus tomentosa*, which are exclusively conserved in Salicaceae plants. Among these genes, the member named *PtoRFL30* was discovered, exhibiting specific expression in the stem. Through genetic and physiological analyses of mitochondrial-targeted *PtoRFL30*, we elucidated its role in modulating mitochondrial homeostasis, thereby influencing wood formation through the regulation of auxin levels in the secondary vasculature. Our findings offer initial insights into the collaborative involvement of mitochondria and phytohormones in the development of secondary vasculature in woody plants.

## Results

### *PtoRFL30* is preferentially expressed in the vascular cambium zone

**Figure 1:**
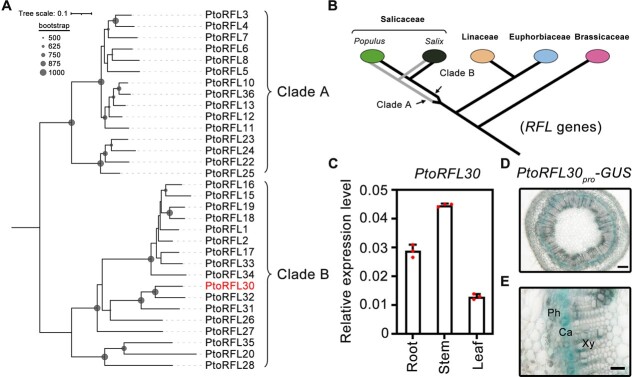
Phylogenetic analysis and expression pattern of *PtoRFL30* in *P. tomentosa*. **A** Phylogenetic relationship of all the *RFL* genes in *P. tomentosa* subgenome A. All the *RFL* sequences used to construct the tree are listed in Supplementary Data Table S1. The JTT + G model and 1000 bootstrap replicates based on amino acid sequences were used to build the phylogenetic tree. Every branch displays support above 500 as a circle dot. **B** Schematic representation of the evolutionary history of the *RFL* genes in Supplementary Data Fig. S1. **C**  *PtoRFL30* relative expression level in various *P. tomentosa* plant tissues as assessed by RT–qPCR. The dots represent the values of each biological replicate. Error bars represent ± standard deviation; *n* = 3. **D**, **E** Histological staining of the stem from GUS reporter lines driven by the promoter of *PtoRFL30*. The seventh internodes of 3-month-old *P. tomentosa* plants were cross-sectioned for GUS staining. Ca, cambium; Ph, phloem; Xy, xylem. Scale bars: 500 μm (**D**); 200 μm (**E**).

To elucidate the evolutionary relationships among *RFL* family genes in poplar and other angiosperm species, we identified *RFL* genes from *A. thaliana* (25), *P. tomentosa* (32), *P. trichocarpa* (34), *Salix purpurea* (61), *Linum usitatissimum* (22), and *Manihot esculenta* (134) (Supplementary Data Table S1). Phylogenetic analysis revealed that the evolution of *RFL* genes in the Salicaceae occurred after Salicaceae divergence (Supplementary Data Fig. S1). Although most *RFL* genes within the Salicaceae form orthologous clusters, a subset of genes in *S. purpurea* forms specific paralogous clusters (Supplementary Data Fig. S1). This suggests that the *RFL* gene family has not yet reached complete evolution and expansion within the Salicaceae. In *P. tomentosa*, 32 *RFL* genes were identified and clustered into two clades (Fig. 1A). Clade A is exclusive to the Salicaceae, while the genes in Clade B share similarities in origin with the *RFL* genes of other species in the Malpighiales (Supplementary Data Fig. S1 and B).

The expression profiles of all *RFL* genes in various tissues of *Populus* were characterized to investigate their potential involvement in wood development (Supplementary Data Fig. S2A). Among these, a member of Clade B, named *PtoRFL30*, exhibited significantly higher expression levels in the stem compared with other tissues (Supplementary Data Fig. S2A and Fig. 1C). Protein structure prediction via the TPRpred online tool (https://toolkit.tuebingen.mpg.de) indicated that *PtoRFL30* encoded a classic PPR protein harboring 14 typical P-type PPR motifs (Supplementary Data Fig. S3). Furthermore, we assessed co-expression patterns of poplar genes in the secondary vasculature by weighted correlation network analysis (WGCNA) using the datasets from the AspWood database [44] (Supplementary Data Fig. S2B). It was found that the co-expression module containing *PtoRFL30* displayed a stronger association with the cambium compared with other tissues (Supplementary Data Fig. S2C). Besides, the *GUS* reporter driven by the *PtoRFL30* promoter in transgenic poplar verified its expression in the cambium zone within the stem (Fig. 1D and E). These findings suggest that *PtoRFL30* is likely involved in the regulation of vascular cambial activity during wood formation.

### *PtoRFL30* negatively regulates vascular cambial activity and wood formation

To determine the physiological role of *PtoRFL30* in wood formation, we conducted both overexpression (OE) and RNA interference (RNAi)-based knockdown experiments targeting *PtoRFL30*. By detecting the *PtoRFL30* expression levels in transgenic plants, two overexpressing lines (L1 and L4) with considerably higher *PtoRFL30* gene expression were chosen for further analysis (Supplementary Data Fig. S4A). The *PtoRFL30*-OE lines displayed reduced plant height, internode number, and stem diameter compared with the wild-type (WT) plants (Supplementary Data Fig. S4A, B, and E–G). Conversely, the opposite phenotype was observed in *PtoRFL30*-RNAi transgenic poplar (L1 and L6), where *PtoRFL30* expression levels were decreased (Supplementary Data Fig. S4C–G).

**Figure 2:**
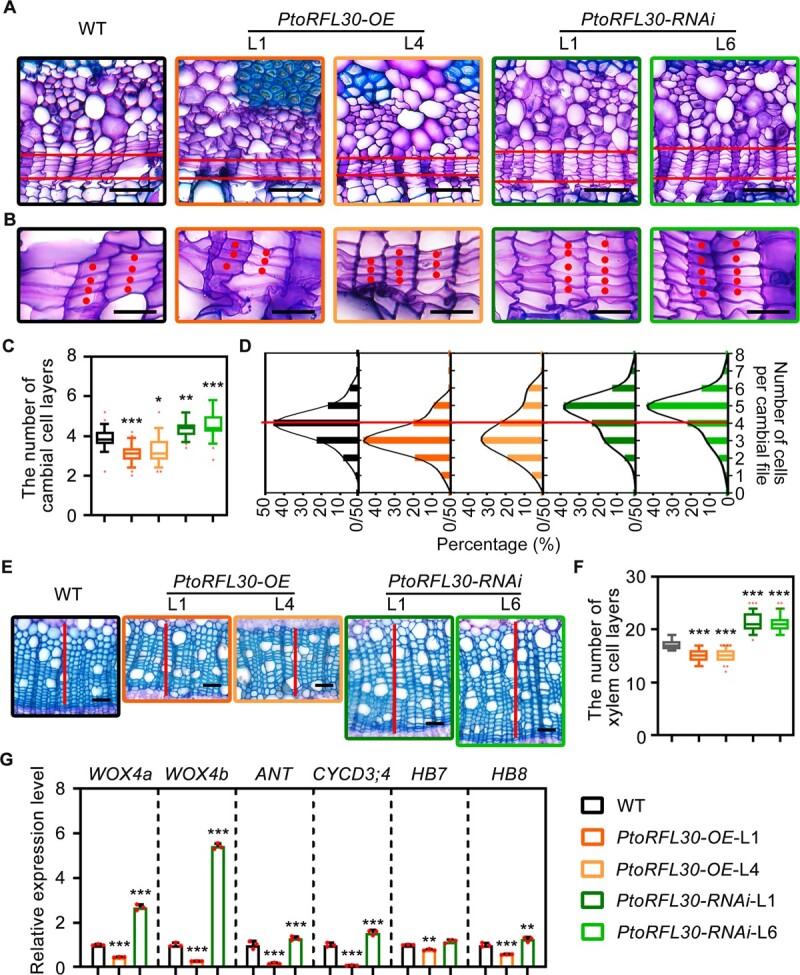
PtoRFL30 regulates vascular cambial activity and xylem development-related phenotypes in *P. tomentosa*. **A**, **B** Phenotype associated with vascular cambial cell proliferation in *PtoRFL30*-OE, *PtoRFL30*-RNAi, and WT transgenic lines. The images were captured on toluidine blue-stained cross-sections of the eighth internode of 3-month-old plants. **A** The areas between lines represent cambial zones. **B** Cambial cells in the same cell file are represented by dots. Scale bars: 50 μm (**A**); 20 μm (**B**). **C** Cambial cell layer number in each file of WT, *PtoRFL30*-OE, and *PtoRFL30*-RNAi transgenic lines. **D** Frequency distributions of cell numbers in each cambial cell file in the stems of WT, *PtoRFL30*-OE, and *PtoRFL30*-RNAi transgenic lines. The WT frequency curve’s highest value is shown by the line. **E** Detailed observation of secondary xylem development of WT, *PtoRFL30*-OE and *PtoRFL30*-RNAi transgenic lines. Lines represent xylem zones. Scale bar: 50 μm. **F** Number of xylem cell layers in stems of WT, *PtoRFL30*-OE, and *PtoRFL30*-RNAi corresponding to **E**. **G** Expression levels of wood formation regulators *WOX4a*, *WOX4b*, *ANT*, *CYCD3;4*, *HB7*, and *HB8* of WT, *PtoRFL30*-OE, and *PtoRFL30*-RNAi transgenic lines. Dots represent the values of biological replicates. Error bars show ± standard deviation. Significant differences from WT are indicated by asterisks (one-way ANOVA, pairwise comparisons, Dunnett’s test): *P < 0.05; **P < 0.01; ***P < 0.001; *n* = 24 (**C**), 40 (**F**), and 3 (**G**).

Vascular development observation in both WT and transgenic plants revealed that the vascular cambium typically consists of four cell layers. However, the *PtoRFL30*-OE and -RNAi transgenic lines exhibited approximately three and five cell layers, respectively, in most of the vascular cambial files (Fig. 2A–D). The variation in the number of cambial cell layers between WT and transgenic plants indicated that PtoRFL30 inhibits cambial activity. Overexpression of *PtoRFL30* led to repression of secondary xylem development, while transgenic plants with reduced *PtoRFL30* expression levels showed a significant increase in the number of secondary xylem cell layers (Fig. 2E and F).

In addition, quantitative PCR (qPCR) was employed to assess the expression of key genes during wood development. The results indicated that *PtoRFL30* overexpression suppresses the transcription of genes associated with cambial activity (*WOX4a*, *WOX4b*, *ANT*, and *CYCD3;4*) [45] and secondary xylem differentiation (*HB7* and *HB8*) [46]. In contrast, the expression levels of these genes were significantly upregulated in *PtoRFL30*-RNAi lines (Fig. 2G). These findings suggest that PtoRFL30 represses vascular cambial activity and wood formation.

### *PtoRFL30* modulates mitochondrial homeostasis

**Figure 3:**
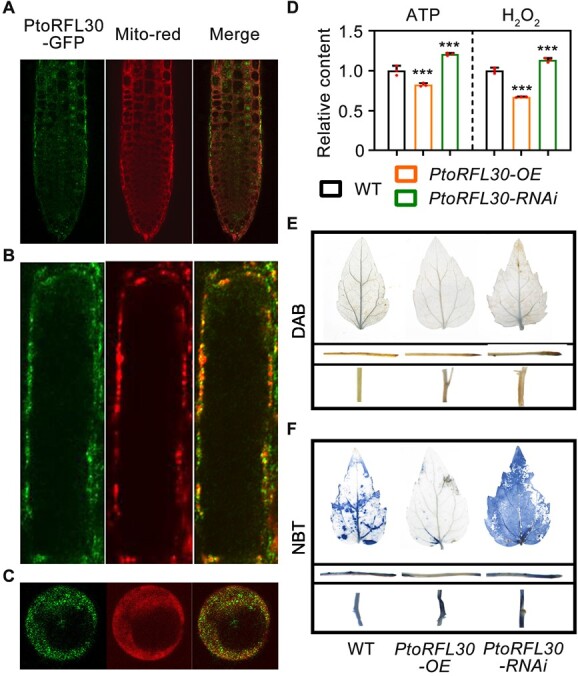
PtoRFL30-mediated changes in mitochondrial state. **A**–**C** Mitochondrial location of PtoRFL30 protein was found in the roots (**A**), root cells (**B**), and root protoplasts (**C**) of transgenic *Arabidopsis* that were carrying ectopically expressed *PtoRFL30-GFP.* The mitochondrial dye Mito-tracker Red (Mito-red) released fluorescence, which confocal microscopy was used to detect. **D** Quantitative measurements of ATP and H_2_O_2_ in the stems of 3-month-old *PtoRFL30*-RNAi, *PtoRFL30*-OE, and WT. Error bars show ± standard deviation. Significant differences from the WT are indicated by asterisks (one-way ANOVA, pairwise comparisons, Dunnett’s test): ***P < 0.001; *n* = 3. (**E**, **F**) 3,30-Diaminobenzidine (DAB) (**E**) and nitrotetrazolium blue chloride (NBT) (**F**) staining of leaves, roots, and stems of WT, *PtoRFL30*-RNAi, and *PtoRFL30*-OE plants.

RFL proteins are typically localized in chloroplasts or mitochondria, exerting influence over the expression of organellar transcripts [15, 47]. In order to ascertain its subcellular localization, we introduced PtoRFL30 fused with a green fluorescent protein (GFP) tag into *Arabidopsis*. The fluorescence signals of PtoRFL30-GFP overlapped with the red fluorescence emitted by mitochondrial-specific dyes (Mito-red) in *Arabidopsis* root cells and isolated root protoplasts (Fig. 3A–C), indicating that PtoRFL30 targets mitochondria.

Mitochondria play a crucial role in cellular respiration, serving as the main source of ATP and ROS. Therefore, ATP and ROS levels are commonly utilized as key biochemical markers to assess mitochondrial function [48]. To confirm the impact of PtoRFL30 proteins on mitochondrial function, we measured the content of ROS and ATP in *PtoRFL30* transgenic plants. In comparison with WT, the ATP content decreased by ~30% in *PtoRFL30*-OE plants, while it increased by >120% in *PtoRFL30*-RNAi lines (Fig. 3D). The content of H_2_O_2_ in *PtoRFL30* transgenic plants also exhibited a significant change of a similar magnitude (Fig. 3D). Additionally, specific dyes for H_2_O_2_ (3,30-diaminobenzidine, DAB) and O_2_^−^ (nitrotetrazolium blue chloride, NBT) were used to stain various tissues of poplar. The results demonstrated that the accumulation of ROS (H_2_O_2_ and O_2_^−^) in leaves, roots, and stems of *PtoRFL30*-OE lines was lower than that of WT, whereas the accumulation level in *PtoRFL30*-RNAi plants was higher (Fig. 3E and F). Furthermore, *PtoRFL30*-OE and -RNAi lines showed variable expression levels of various mitochondrial-encoded genes when compared with WT (Supplementary Data Fig. S5), suggesting that PtoRFL30 functionally modulates mitochondria. These findings indicate that variations in *PtoRFL30* expression levels compromise mitochondrial homeostasis.

### *PtoRFL30*-mediated mitochondrial homeostasis is necessary for wood formation

To investigate whether mitochondrial homeostasis is involved in wood growth, we treated WT plants with two mitochondrial function inhibitors, rotenone and antimycin A, which inhibit complexes I and III of the electron transfer chain in mitochondria, respectively. Both inhibitor treatments resulted in a significant reduction in the cell layer number of vascular cambium and secondary xylem (Supplementary Data Fig. S6A–E). Additionally, the expression levels of key genes related to vascular cambial cell division and secondary xylem differentiation were inhibited, especially under antimycin A treatment (Supplementary Data Fig. S6F). This suggests that maintaining mitochondrial homeostasis is essential for wood formation.

**Figure 4:**
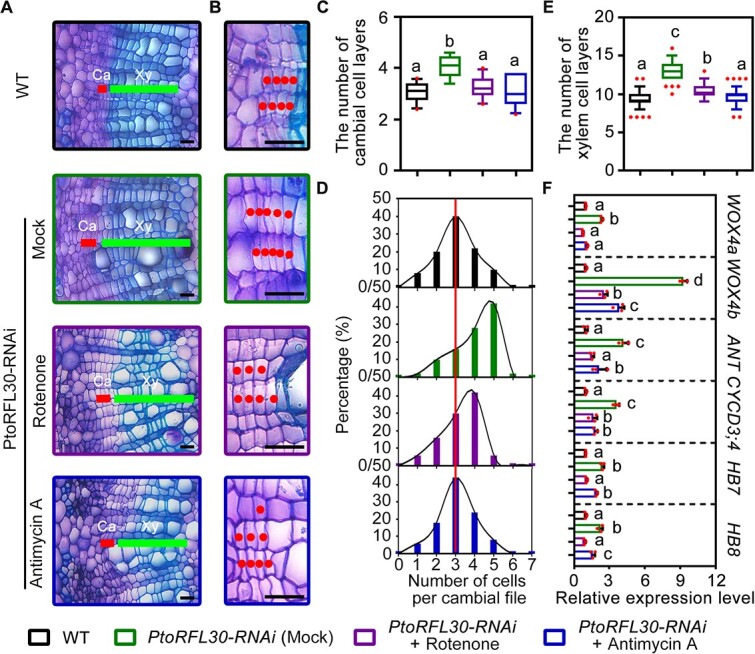
Rescue of wood formation phenotypes resulting from reduced expression of *PtoRFL30* by mitochondrial function inhibitors. **A**, **B** Detailed observation of toluidine blue-stained cross-sections of the eighth internode of 3-month-old WT and *PtoRFL30*-RNAi transgenic lines under treatment with mitochondrial function inhibitors (10 μM rotenone and 50 μM antimycin A). Dots in **B** represent cambial cells in the same cell files. Ca, cambium; Xy, xylem. Scale bars: (**A**) 50 μm; (**B**) 20 μm. **C** Cambial cell layer number in each cell file of WT and *PtoRFL30*-RNAi transgenic lines treated with mitochondrial function inhibitors. **D** Cell number frequency distributions in cambial cell files in the stems of WT and *PtoRFL30*-RNAi transgenic lines treated with mitochondrial function inhibitors. The peak value of the WT frequency curve is indicated by the line. **E** Number of xylem cell layers in stems of WT and *PtoRFL30*-RNAi transgenic lines treated with mitochondrial function inhibitors, corresponding to **A**. **F** Expression levels of wood formation regulators *WOX4a*, *WOX4b*, *ANT*, *CYCD3;4**,** HB7*, and *HB8* of WT and *PtoRFL30*-RNAi transgenic lines treated with mitochondrial function inhibitors. Dots represent the values of each biological replicate. Error bars show ± standard deviation. Significant differences are shown by letters above the error bars (one-way ANOVA followed by pairwise comparisons using Tukey’s test); *n* = 10 (**C**); *n* = 49 (**E**); *n* = 3 (**F**).

Furthermore, we applied rotenone and antimycin A to *PtoRFL30*-RNAi transgenic plants. It was found that the expression level of mitochondrial-encoded genes in *PtoRFL30*-RNAi transgenic plants was restored under treatment with mitochondrial function inhibitors (Supplementary Data Fig. S7). Observation of the wood developmental phenotype showed that the number of vascular cambial cell layers and secondary xylem cell layers in lines treated with rotenone was significantly reduced compared with untreated *PtoRFL30*-RNAi transgenic plants (Fig. 4A–E), with little variation in the expression level of genes related to wood development (except for *WOX4b* and *CYCD3;4*, which did not return to the expression level in WT) (Fig. 4F). Antimycin A treatment restored excess wood development in *PtoRFL30*-RNAi plants to a state similar to WT, including the expression level of these key genes (Fig. 4). This indicates that PtoRFL30-mediated mitochondrial homeostasis is crucial for maintaining normal wood development.

### *PtoRFL30* inhibits accumulation of auxin in the secondary vasculature

**Figure 5:**
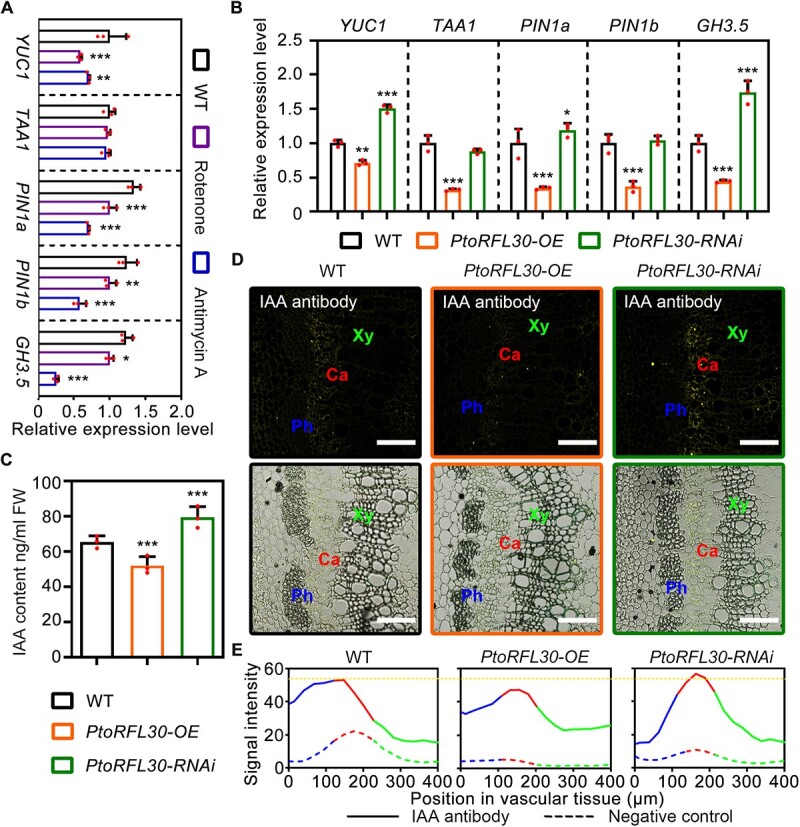
Auxin accumulation is altered by PtoRFL30-mediated mitochondrial homeostasis during wood formation. **A**, **B** Expression levels of *YUC1*, *TAA1*, *PIN1a*, *PIN1b*, and *GH3.5*, which are genes related to auxin homeostasis, in stems of WT under treatment with mitochondrial function inhibitors (10 μM rotenone or 50 μM antimycin A) (**A**) and in *PtoRFL30*-OE and *PtoRFL30*-RNAi transgenic lines (**B**). **C** Content of auxin (IAA) in stems of WT, *PtoRFL30*-OE, and *PtoRFL30*-RNAi transgenic lines. Dots represent values of each biological replicate. Error bars show ± standard deviation. Significant differences from WT are indicated by asterisks (one-way ANOVA, pairwise comparisons, Dunnett’s test): *P < 0.05; **P < 0.01; ***P < 0.001; *n* = 3. **D** Immunofluorescence of auxin (IAA) in secondary vasculature of WT, *PtoRFL30*-OE, and *PtoRFL30*-RNAi transgenic lines. Ph, phloem; Ca, cambium; Xy, xylem. Scale bar: 100 μm. **E** Intensity analysis of immunofluorescent signals of auxin (IAA) in secondary vasculature of WT, *PtoRFL30*-OE, and *PtoRFL30*-RNAi transgenic lines. Fluorescence intensity in the negative control is indicated by the dashed line. The highest value of fluorescence intensity in WT is shown by the dotted line.

Previous studies have established that auxin transport and homeostasis are essential for microRNA476a–RFL module-mediated mitochondrial homeostasis, leading to adventitious root formation [38]. Therefore, we examined the expression levels of genes associated with auxin homeostasis in response to mitochondrial function inhibitor treatment and *PtoRFL30* transgenic plants to investigate whether PtoRFL30 influences auxin accumulation during wood formation. Under mitochondrial function inhibitor treatment, the expression levels of the auxin biosynthesis gene (*YUC1*), polar transport genes (*PIN1a*, *PIN1b*), and primary auxin-responsive gene (*GH3.5*) were significantly reduced (Fig. 5A). Simultaneously, *PtoRFL30* overexpression markedly suppressed the expression of auxin biosynthesis and transport genes (Fig. 5B). A decrease in auxin signaling *in vivo* was indicated by the downregulation of the primary auxin-responsive gene *GH3.5*, as further supported by testing the auxin content of the poplar stem (Fig. 5B and C). In contrast to *PtoRFL30*-OE plants, *PtoRFL30*-RNAi plants exhibited upregulation of auxin homeostasis-related genes and increased auxin accumulation (Fig. 5B and C). Immunofluorescence imaging using the antibody against IAA revealed that PtoRFL30 altered auxin content in the stem but did not affect the auxin distribution pattern in vasculature (Fig. 5D and E).

### *PtoRFL30* regulates wood formation by modulating auxin homeostasis

**Figure 6:**
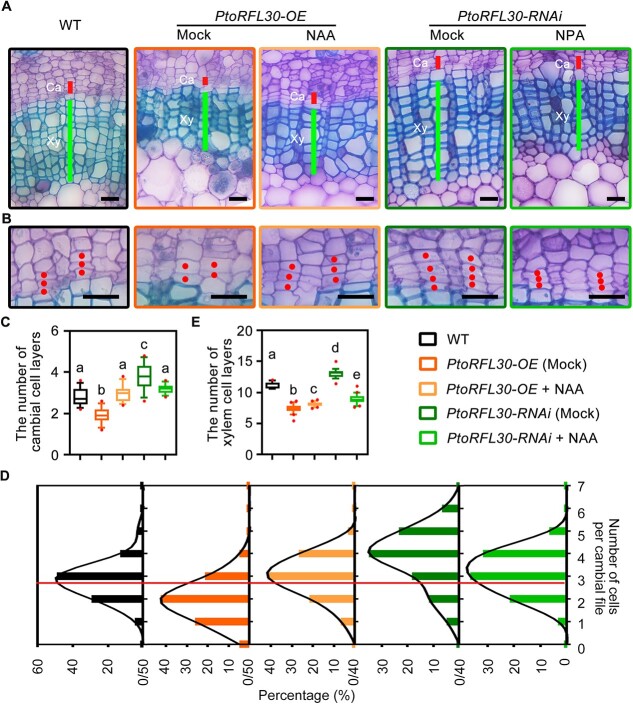
Wood formation phenotypes resulting from auxin and auxin transport inhibitor treatment in *PtoRFL30*-related transgenic plants. **A**, **B** Anatomical sections of the eighth internode of 3-month-old wild-type (WT), *PtoRFL30*-OE transgenic lines treated with auxin (0.1 μM NAA), and *PtoRFL30*-RNAi transgenic lines treated with auxin transport inhibitor (5 μM NPA), stained with toluidine blue. **B** Dots represent cambial cells in the same cell files. Ca, cambium; Xy, xylem. Scale bars: (**A**) 50 μm; (**B**) 20 μm. **C** Cambial cell layer number in each cell file of WT and *PtoRFL30*-OE transgenic lines under 0.1 μM NAA treatment and *PtoRFL30*-RNAi transgenic lines under 5 μM NPA treatment. **D** Frequency distributions of cell numbers in cambial cell files in the stems of WT, *PtoRFL30*-OE transgenic lines under 0.1 μM NAA treatment and *PtoRFL30*-RNAi transgenic lines under 5 μM NPA treatment. The peak location of the WT frequency curve is indicated by the red line. **E** Number of xylem cell layers in stems of WT, *PtoRFL30*-OE transgenic lines under 0.1 μM NAA treatment, and *PtoRFL30*-RNAi transgenic lines under 5 μM NPA treatment, corresponding to (**A**). Error bars show ± standard deviation. Significant differences are indicated by letters above the error bars (one-way ANOVA followed by Tukey’s test for pairwise comparisons); *n* = 12 (**C**); *n* = 20 (**E**).

Excessive *PtoRFL30* expression and mitochondrial function inhibitors (antimycin A and rotenone) disrupt mitochondrial homeostasis, impeding vascular cambial activity and secondary xylem differentiation, contrary to the known influence of auxin on wood formation in poplar [45, 46]. Conversely, exogenous auxin supplementation mitigates the effects of mitochondrial function inhibitors, delaying wood development (Supplementary Data Fig. S8). To further validate the necessity of auxin homeostasis for PtoRFL30-mediated wood development,*PtoRFL30*-OE transgenic plants were treated with NAA. NAA treatment partially restored the defects in secondary vascular development caused by *PtoRFL30* overexpression compared with the mock control (Fig. 6). Auxin treatment restored the number of vascular cambial cell layers in *PtoRFL30*-OE transgenic plants to WT levels (Fig. 6A–D), while the number of xylem cell layers increased but did not reach the WT level (Fig. 6A and E). In *PtoRFL30*-RNAi plants, blocking polar auxin transport with NPA significantly reduced the number of cell layers in the vascular cambium and secondary xylem, leading to defects in wood development (Fig. 6). Together, these findings indicate the dependence of PtoRFL30-mediated wood formation on auxin homeostasis in secondary vasculature.

## Discussion

**Figure 7:**
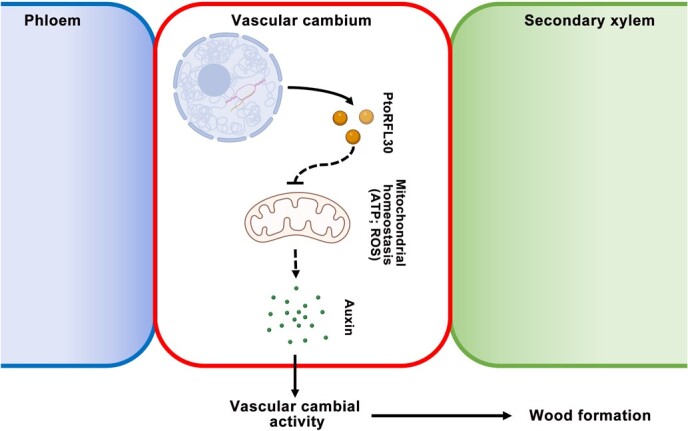
Model for mitochondrial homeostasis mediated by PtoRFL30 during wood formation in poplar. During wood formation, nuclear-encoded protein PtoRFL30 targets mitochondria, and affects mitochondrial functional homeostasis. Furthermore, PtoRFL30-mediated mitochondrial signaling regulates vascular cambial activity by altering auxin accumulation in the secondary vasculature.

RFL proteins, recognized as sequence-specific RNA-binding proteins crucial for CMS restoration [7, 9], have been implicated in diverse functions throughout the plant life cycle, yet their involvement in secondary vascular development remains unexplored [25, 49]. This study unveils PtoRFL30 as a mitochondrial-targeted PPR protein in poplar, demonstrating specific expression in the vascular cambium and a negative regulatory role in wood development by influencing mitochondrial homeostasis. Importantly, we elucidate the PtoRFL30-mediated mitochondrial regulation of wood formation, emphasizing its dependency on auxin accumulation within the secondary vasculature (Fig. 7).

The *RFL* gene family, well known for its rapid evolution, engages in a molecular ‘arms race’ between the mitochondrial and nuclear genomes [9, 20, 21]. This swift evolution is attributed to the emergence of new *PPR* genes in response to evolving infertility-inducing genes. The intricate interplay between the mitochondrial and nuclear genomes has been recognized as a dynamic process [20, 50, 51]. Notably, *RFL* genes exhibit species specificity, adapting to random mitochondrial genome mutations, with each *Rf* gene recognizing the transcript sequence of a specific mitochondrial gene [8, 16, 19]. Our study reveals that rapid evolution persisted after Salicaceae divergence, leading to the emergence of distinct *RFL* genes in Salicaceae plant genomes (Fig. 1B, Supplementary Data Fig. S1). However, in dioecious Salicaceae plants, where cross-pollination circumvents the negative effects of CMS on reproduction, the presence of *RFL* genes may not be as pronounced. Previous studies have demonstrated the regulatory role of poplar *RFL* genes in adventitious root formation through a mitochondria-dependent pathway [38]. Building on this, we demonstrate that PtoRFL30 regulates wood formation in *Populus* through mitochondrial regulation, intertwined with auxin homeostasis. Understanding the specific functions of *RFL* genes in dioecious species unveils novel insights into how mitochondrial signaling orchestrates plant growth and development.

Mitochondrial function is tightly integrated into metabolism and biosynthetic processes and is also crucial for signaling and responding to stresses [52–55]. In plants, the levels of signals and energy metabolism are used to determine the mitochondrial functional state [48]. The levels of mitochondrial energy metabolites ATP (or the ATP/ADP ratio) and NAD/NADH are known to directly or indirectly affect plant development [56]. While ROS serve as mitochondrial retrograde signals, their excessive production poses a dual role, regulating diverse biological processes yet inducing oxidative stress [40, 42, 43, 57, 58]. In poplar, mitochondrial signaling through ROS has been implicated in responses to biotic and abiotic stress [59–61]. However, a delicate balance must be maintained, as heightened ROS levels can lead to cellular damage. The importance of sustaining mitochondrial functional homeostasis is underscored by its role in secondary vascular development (Supplementary Data Fig. S6). We further identified that the PtoRFL30 protein is localized in mitochondria and regulates the levels of ATP and ROS in poplar (Fig. 3). The secondary vascular developmental phenotype caused by PtoRFL30 deficiency was restored by altering the mitochondrial state (Fig. 4). All of these findings point to a role for PtoRFL30 in preserving mitochondrial homeostasis throughout the growth of wood.

It is generally believed that ROS and Ca^2+^ are related to the mitochondrial retrograde signaling cascade [62–64]. Given that ROS are generated through the mitochondrial respiratory electron transport chain (mETC), they often serve as a messenger for mitochondrial metabolic status, interlinked with key mitochondrial metabolites like ATP, acetyl-CoA, TCA cycle intermediates, and NAD/NADH [48, 65, 66]. Despite their crucial roles, ROS presently lack well-defined receptors [38, 48]. While mitochondrial retrograde signaling has undergone extensive exploration in yeast and mammalian cells, the regulatory network governing mitochondrial and nuclear communication during plant development remains an area in need of further refinement. In the course of PtoRFL30-mediated wood development, the perturbation of mitochondrial homeostasis exerts influence on the levels of auxin transport and homeostasis-related gene expression, consequently impacting auxin accumulation within the secondary vasculature (Fig. 5). This suggests a regulatory mechanism wherein nuclear genes undergo transcription under the governance of signaling pathways originating in the mitochondria and translocating to the nucleus.

As *RFL30* encodes a typical P-type PPR protein (Supplementary Data Fig. S3), the possibility cannot be excluded that it still acts during reproductive processes like CMS in gynodioecious horticultural plants. *RFL* genes encode P-type PPR proteins that generally participate in 3′ and 5′ terminal processing, RNA stabilization, cleavage, translation activation, and RNA intron splicing regulation of mitochondrial genes [4, 15]. In order to restore CMS, Rf-PPRs mainly target genes that are toxic to reproductive development in the mitochondrial genome, and restore the fertility of plants by inhibiting their target protein production [16–19]. However, overexpression of *PtoRFL30* exhibits an inhibitory effect on secondary vascular development (Fig. 2), suggesting that its target genes may not be negative regulatory factors for normal plant development. In addition, the regulatory mechanism of PtoRFL30 in wood formation is more likely to contribute to maintaining mitochondrial homeostasis by limiting mitochondrial function and affecting mitochondrial energy metabolism and electron transfer (Fig. 3 and Supplementary Data Fig. S5). Meanwhile, PtoRFL30 utilizes the mitochondrial retrograde signaling pathway to influence endogenous signals (such as auxin) to regulate secondary vascular development (Figs 5 and 7). This is an unrecognized role of RFLs to regulate plant development, which relies on a different mechanism than RFLs to restore CMS. Considering the large number of *RFL* genes in the plant genome and their function in *de novo* root organogenesis and vascular cambium activity, it is likely that RFL proteins represent a group of key regulators in multiple developmental programs of plants, including dioecious horticultural plants. According to this conclusion, *RFL* genes may possess potential roles in regulating important traits of gardening plants, which affect the vegetative growth and reproductive development of plants through diverse mechanisms.

In conclusion, a comprehensive examination of the evolutionary relationships among *RFL* family genes in poplar and other angiosperm species reveals that, despite poplar being dioecious, its genome retains a significant number of *RFL* genes. Within the poplar *RFL* family, *PtoRFL30* exhibits preferential expression in the vascular tissue of the stem, playing a crucial role in upholding mitochondrial functional homeostasis. Moreover, PtoRFL30, through its influence on auxin accumulation in the secondary vasculature, orchestrates mitochondrial states that regulate vascular cambial activity and xylem development. These findings provide insights into the novel role of PtoRFL30 in poplar, highlighting its involvement in coordinating mitochondrial homeostasis and auxin levels during wood formation.

## Materials and methods

### Phylogenetic analysis

All the RFL amino acid sequences used to construct the phylogenetic tree are listed in Supplementary Data Table S1. The JTT + G model and 1000 bootstrap replicates based on amino acid sequences were used to build the phylogenetic tree.

### Weighted correlation network analysis

WGCNA (1.72) was used to identify co-expressed genes according to the protocol [67]. The list and expression data of differentially expressed genes (DEGs) were downloaded from AspWood [44]. WGCNA network construction and module detection were performed with an unsigned type of topological overlap matrix (TOM), a power β of 6, a minimal module size of 30, and a branch merge cut height of 0.15. The sample classification information used for trait association analysis came from AspWood [10].

### Gene cloning and plasmid construction

Both *PtoRFL30*-OE and *PtoRFL30-GFP* plant expression vectors were constructed by Xu *et al*. [38]. The promoter sequence upstream of the *PtoRFL30* was amplified from genomic DNA of *P. tomentosa* and inserted into XcmI-digested *pCXGUS-P* to drive *GUS* expression [68]. The 208-bp specific sequence of *PtoRFL30* and its reverse complementary sequence from cDNA of *P. tomentosa* were constructed into the *PtoRFL30*-RNAi plant expression vector as described previously [69].

### Genetic transformation and growth conditions

The above plant expression vectors were stably transformed into leaf disks from WT *P. tomentosa* by *Agrobacterium*-mediated infiltration as described previously [70]. For phenotypic analysis, seedlings were cultivated in the greenhouse [23–25°C, 16 h light (10 000 lux):8 h dark] for 3 months.

### Cross-sections and GUS staining

Stems of 3-month-old *P. tomentosa* plants were cross-sectioned using a vibrating blade microtome (VT1000s; Leica, Germany). After soaking in precooled acetone for 1 h at −20°C, the cross-sections were stained for 15 min at 37°C in the dark using GUS staining solution [0.5 M Tris, pH 7.0, 10% Triton X-100 with 1 mM X-Gluc (5-bromo-4-chloro-3-indolyl-d-glucuronide)]. The stained cross-sections were imaged under a microscope (BX53; Olympus, Japan).

### RT–qPCR

Total RNA was isolated with the Biospin Plant Total RNA Extraction Kit (Bioflux, China). Then, the PrimeScript RT Reagent Kit with gDNA Eraser (0047A; TaKaRa, China) was used to transcribe RNA into cDNA. RT–qPCR was performed in a Real-Time PCR machine (qTOWER3G; Analytik Jena, Germany) with Hieff qPCR SYBR Green Master Mix (11201ES08; Yeasen, China). The *UBIQUITIN* (*UBQ*) gene of poplar was used as the reference gene when calculating the expression data using the ΔΔCt method. The sequences of primers used are listed in Supplementary Data Table S2.

### Quantification of ATP and H_2_O_2_ contents

The ATP and H_2_O_2_ contents of the eighth internodes from 3-month-old *P. tomentosa* WT and transgenic plants were measured using a hydrogen peroxide assay kit (S0038; Beyotime Biotechnology, China) and an ATP assay kit (S0026; Beyotime Biotechnology, China).

### IAA content quantification

As previously described [71], 0.5 g poplar stem samples of WT and transgenic plants were gathered for IAA purification. Quantification was carried out using an LC-ESI-MS/MS system (4000 Q-Trap; Sciex, USA). As an internal standard, 50 ng of [^13^C_6_] IAA was added to each extraction buffer.

### Immunohistochemical localization of auxin

The procedures previously outlined for sample preparation and immunofluorescence detection were followed [72]. In short, stem cross-sections of 3-month-old WT and transgenic plants were treated with a polyclonal antibody against IAA (Agrisera, Sweden) as a primary antibody. DyLight550-labeled secondary antibody (Abcam, UK) was used to detect the signal on a confocal microscope (TCS SP8; Leica, Germany) with excitation set at 550 nm and emission ranging from 560 to 600 nm.

#### Accession number

The GenBank accession number of the *P. tomentosa* gene *PtoRFL30* is MN242837.

## Supplementary Material

Web_Material_uhae188

## Data Availability

The authors confirm that the all data supporting the findings of this study are available within the article and its supplementary materials.
