# Supplementary figures and images for: Restorer of fertility like 30, encoding a mitochondrion-localized pentatricopeptide repeat protein, regulates wood formation in poplar
Source: Hortic Res. 2024 Jul 15;11(9):uhae188. doi: 10.1093/hr/uhae188 (PMC11377185; doi:10.1093/hr/uhae188)

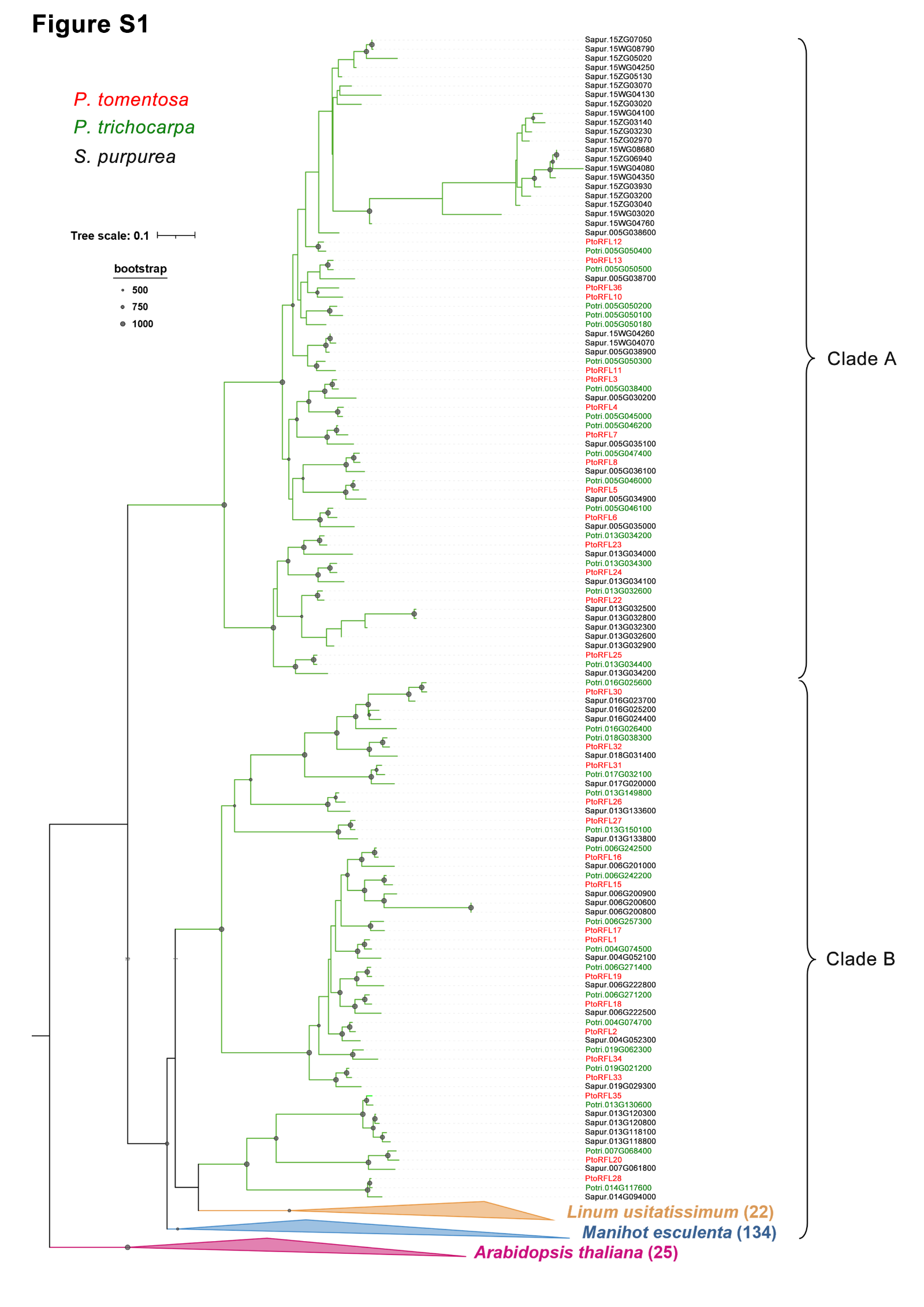

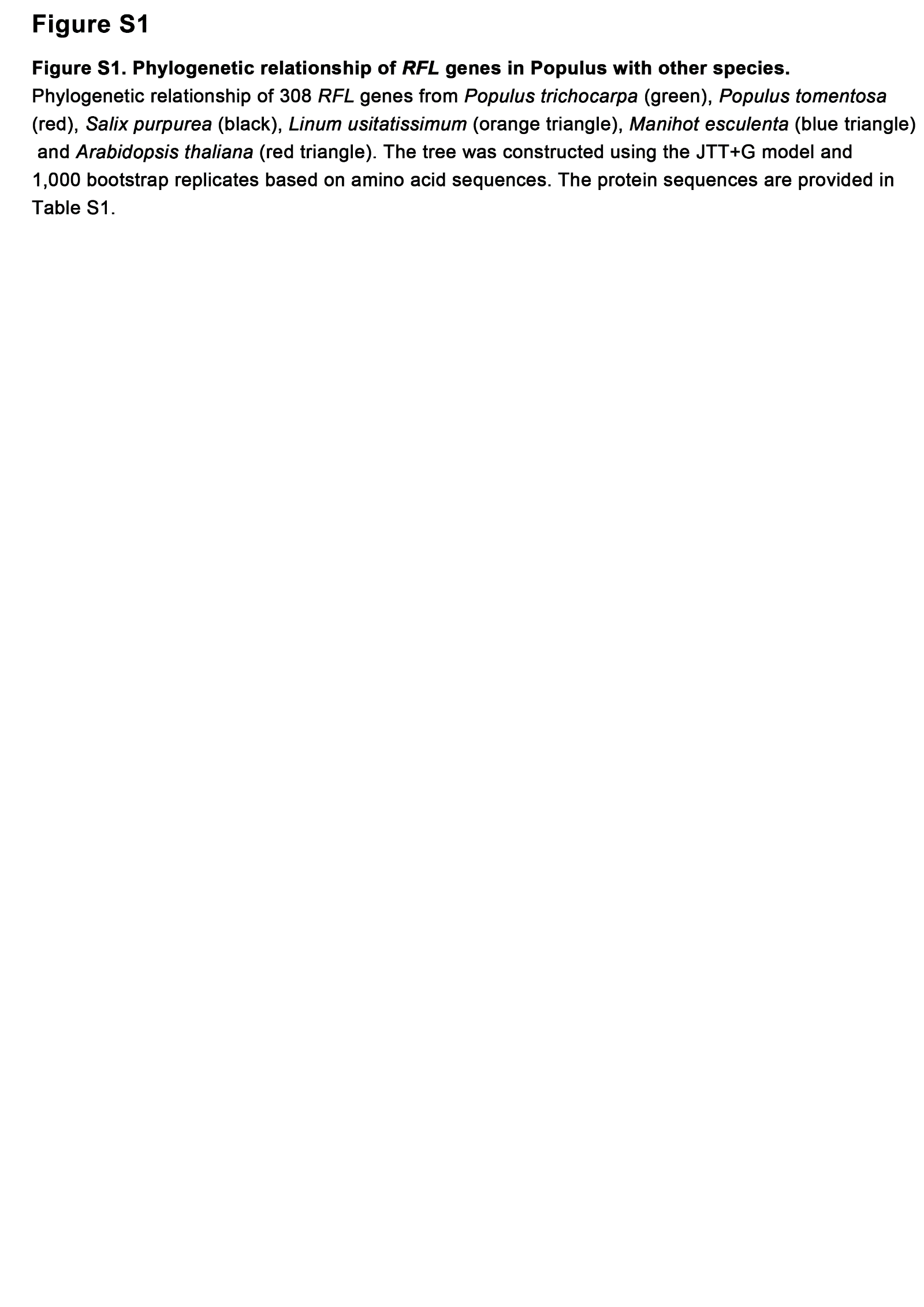

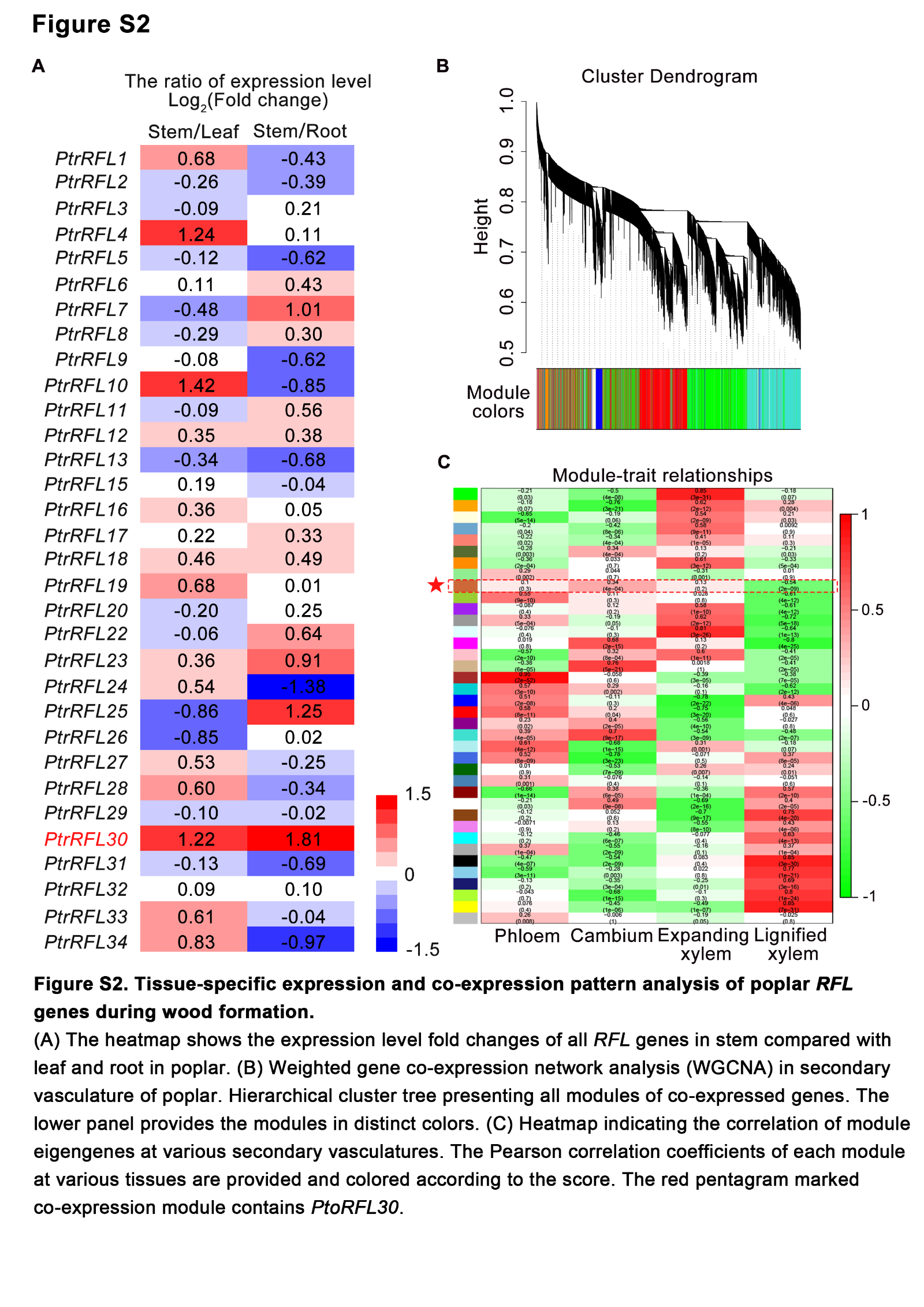

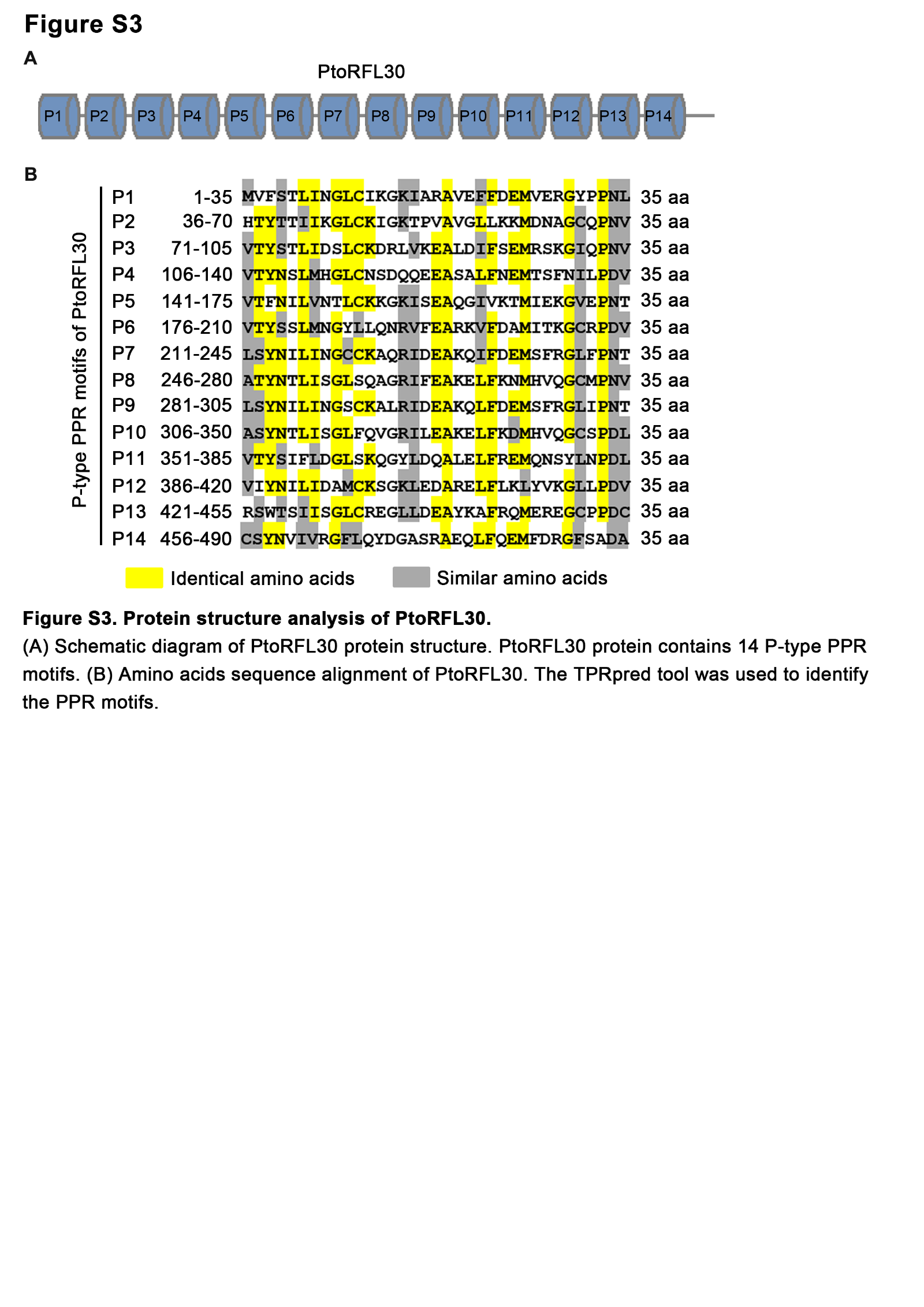

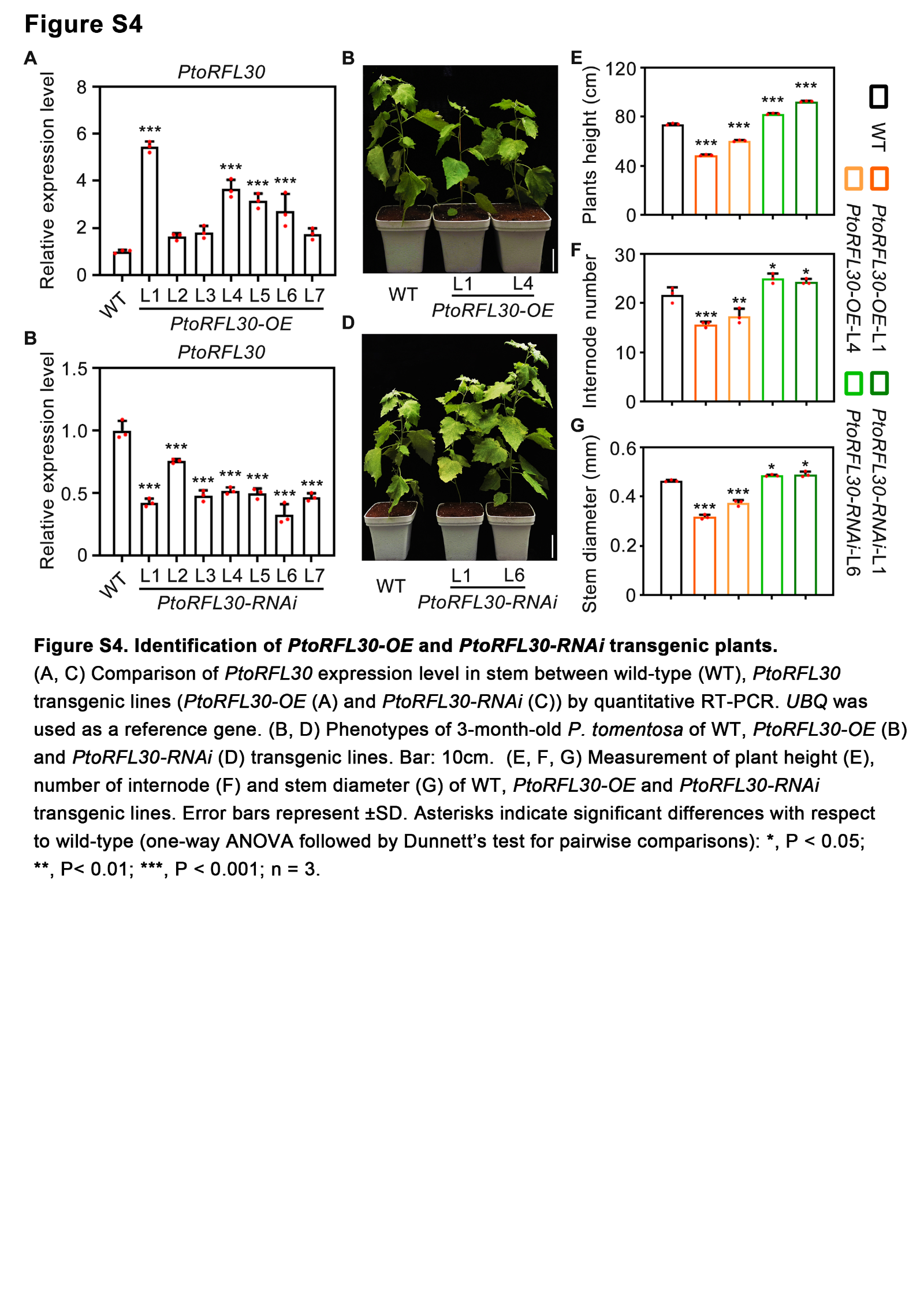

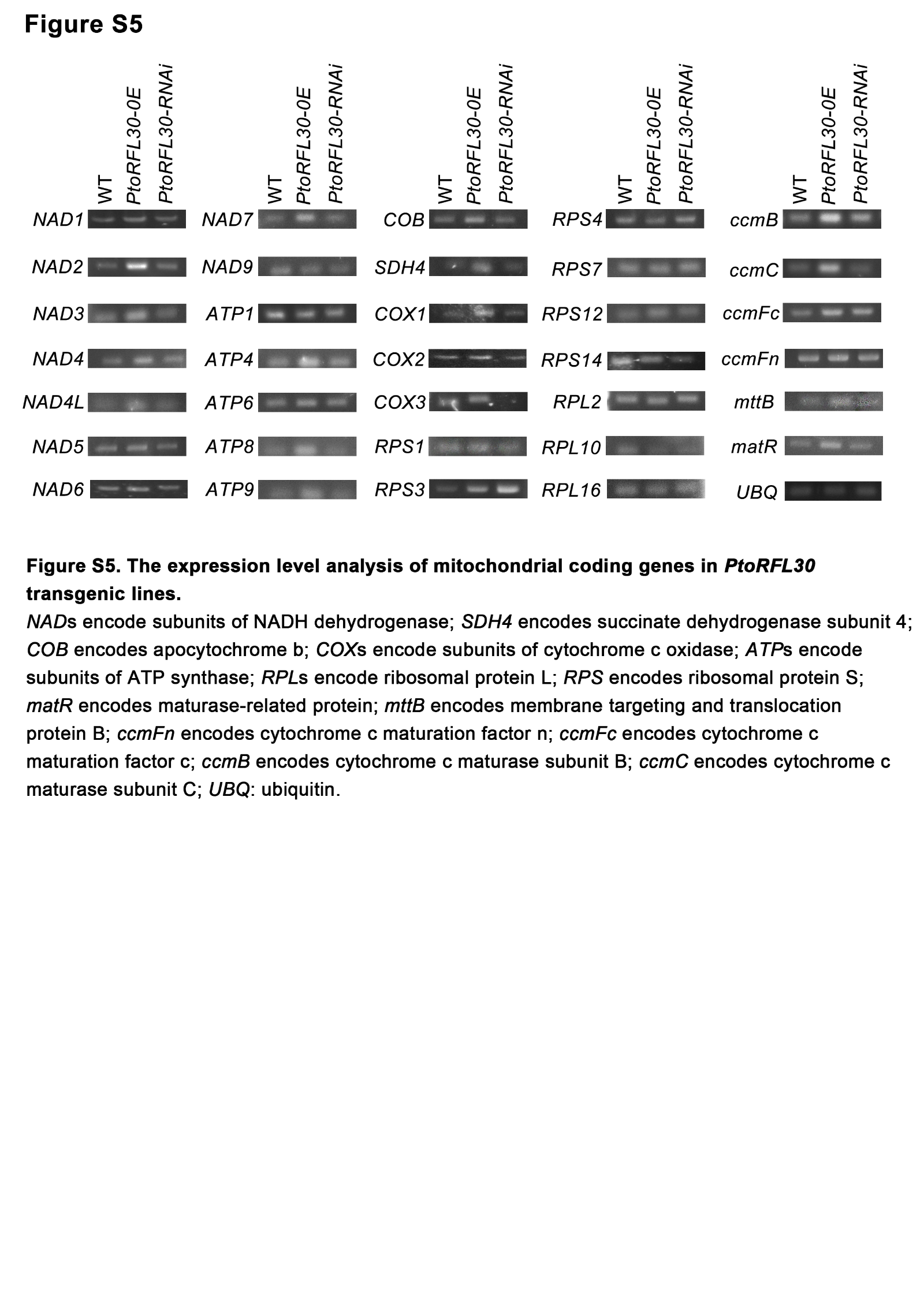

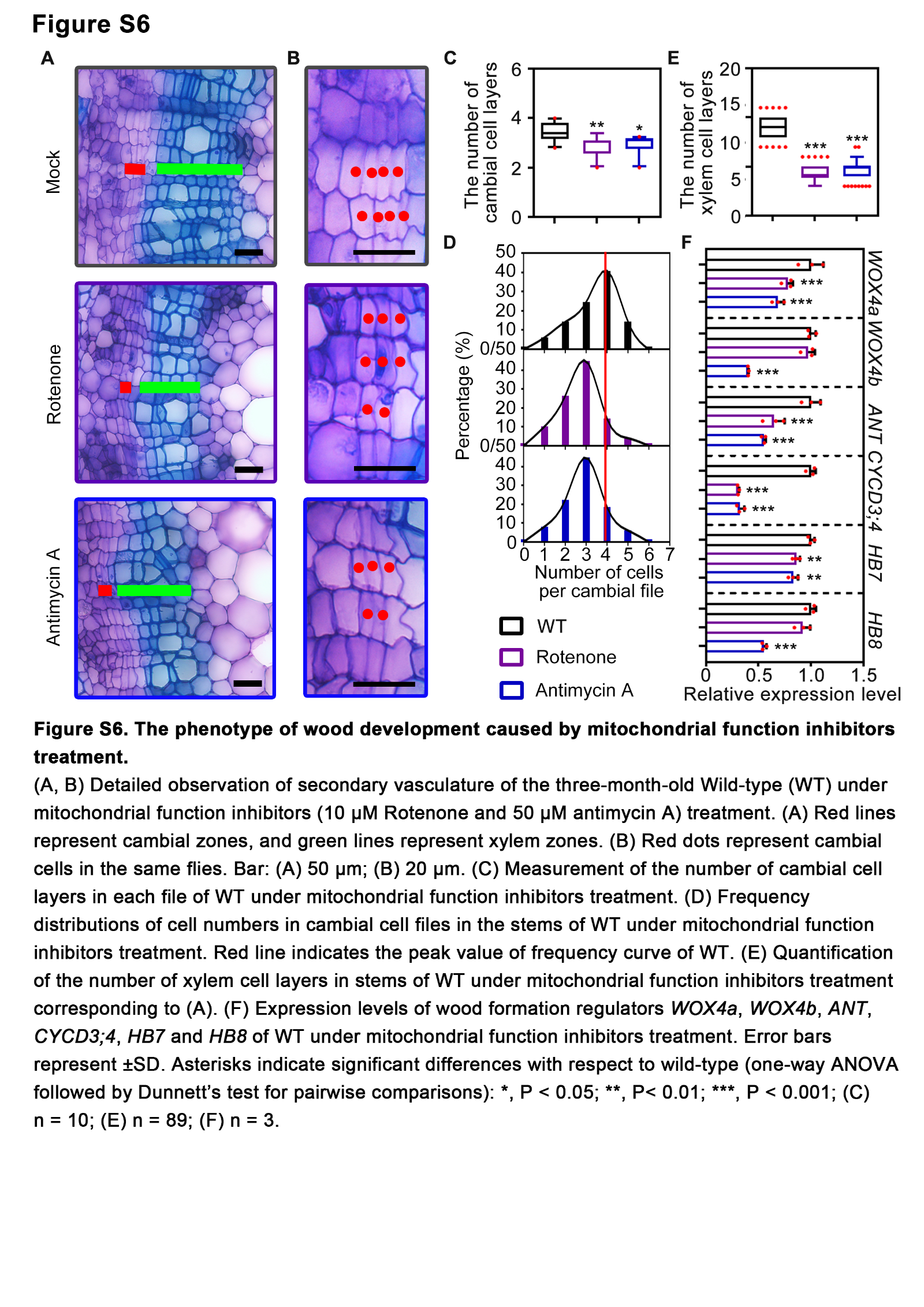

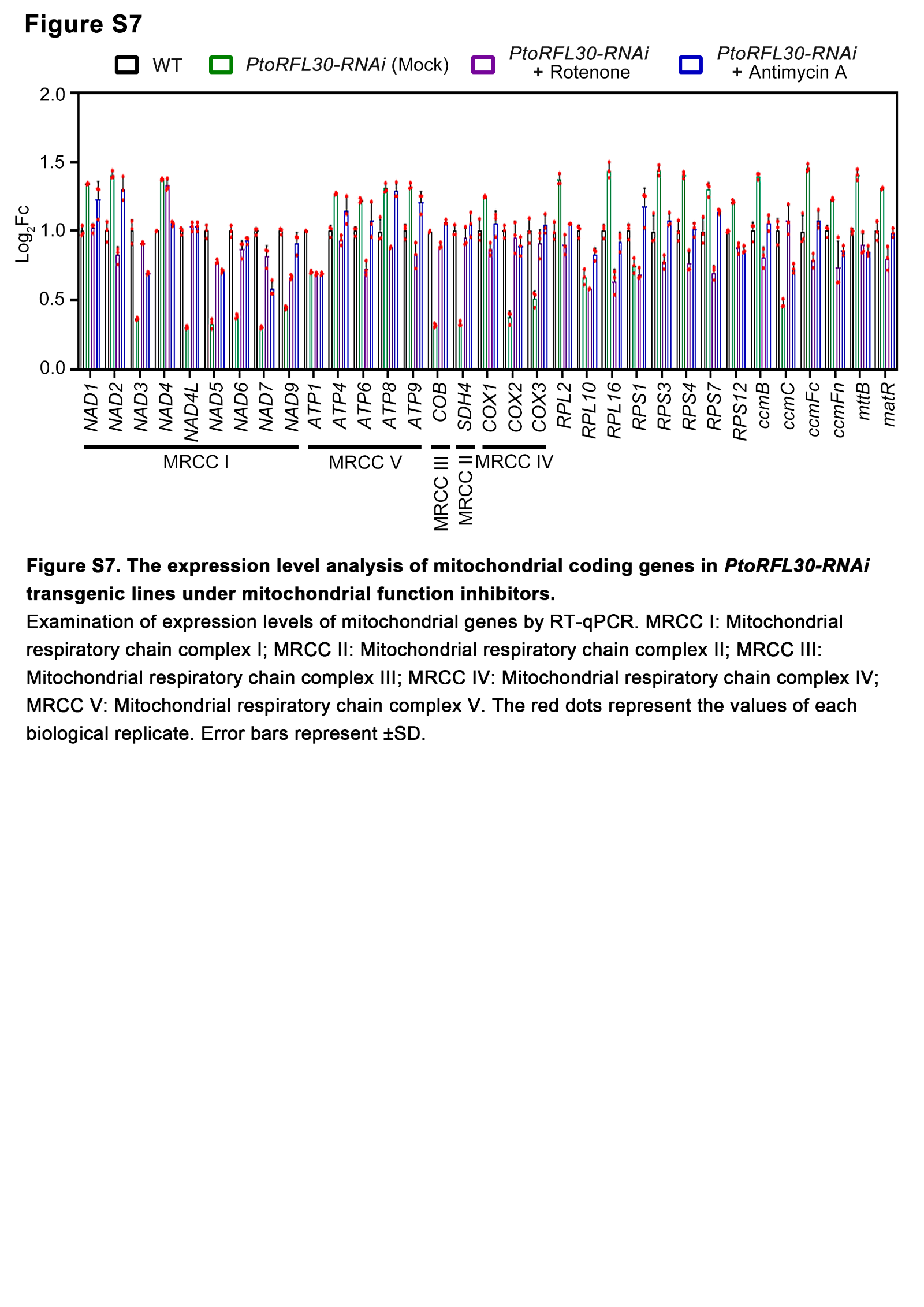

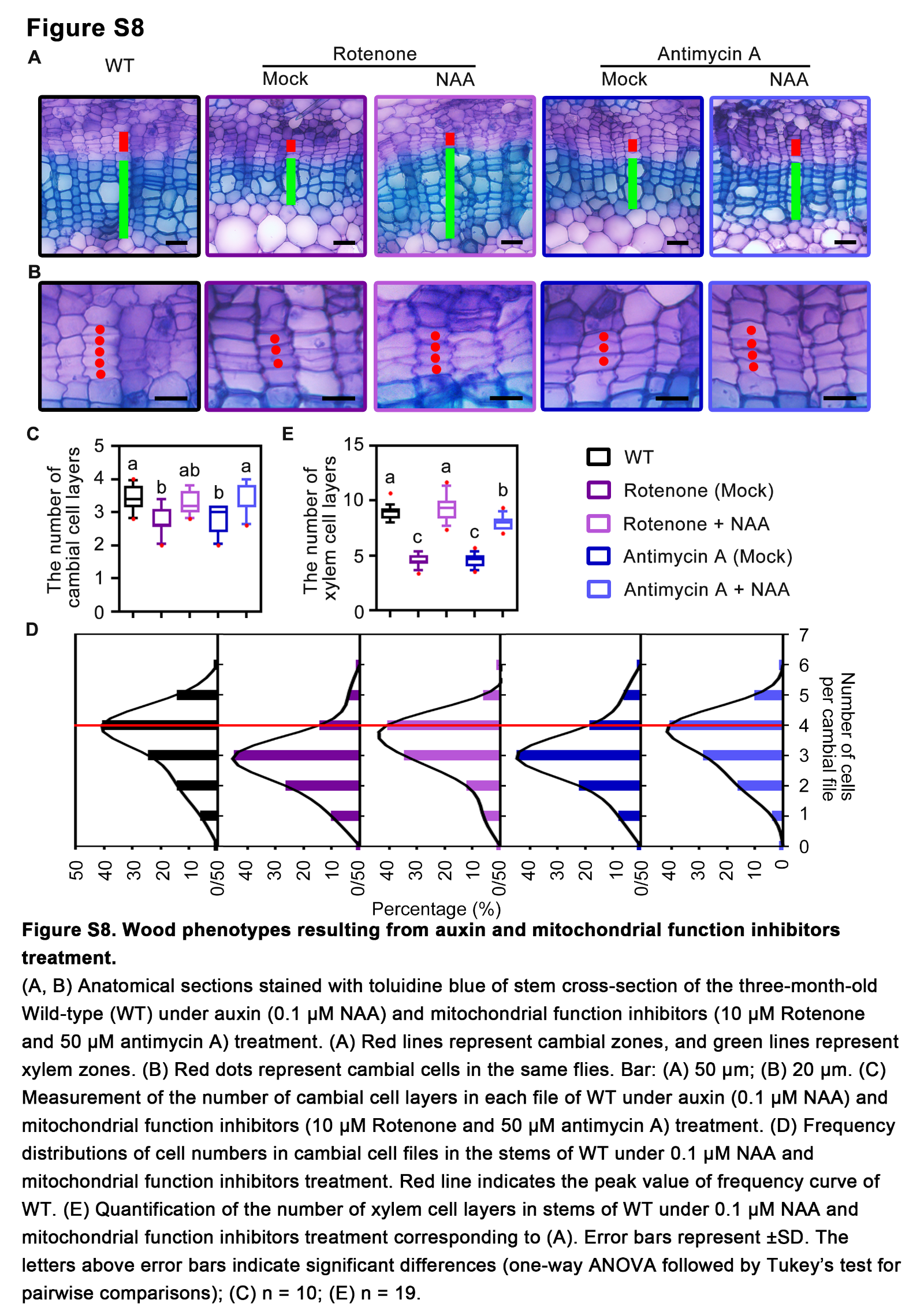

Supplement: Web_Material_uhae188 [file web_material_uhae188.zip › Supplementary Figure.docx]
